# The impact of large core and late treatment trials: An update on the modelled annual thrombectomy eligibility of UK stroke patients

**DOI:** 10.1177/23969873241232820

**Published:** 2024-02-17

**Authors:** Peter McMeekin, Martin James, Christopher I Price, Gary A Ford, Philip White

**Affiliations:** 1 Faculty of Health and Life Sciences, Northumbria University, Newcastle upon Tyne, UK; 2 Peninsula Applied Research Collaboration (PenARC), University of Exeter, Exeter, Devon, UK; 3 Stroke Research Group, Population Health Sciences Institute, Newcastle University, Newcastle upon Tyne, UK; 4 Oxford University Hospitals NHS Foundation Trust and Medical Sciences Division, University of Oxford, Oxford, UK; 5 Translational and Clinical Research Institute, Newcastle University, Newcastle upon Tyne, UK; 6 Newcastle upon Tyne Hospitals NHS Foundation Trust, Newcastle upon Tyne, UK

**Keywords:** Eligibility, late presenters, thrombectomy, ischaemic stroke, large core infarct

## Abstract

**Introduction:**

To support decisions about thrombectomy provision, we have previously estimated the annual UK population eligible for treatment as ∼10% of stroke admissions. Since then, eight further randomised trials that could alter the eligibility rate have reported in 2021-23. We updated our estimates of the eligible population from these trials and other recent studies.

**Patients and methods:**

An updated decision tree describing the EVT eligible population for UK stroke admissions was produced. Decision criteria were derived from the highest level of evidence available. For nodes where no specific RCT data existed, evidence was obtained from the latest systematic review(s) or the highest quality observational data.

**Results:**

We estimate that 15,420 (approximately 15%) of admitted UK stroke patients are now eligible for thrombectomy, or 14,930 if advanced brain imaging using MRI/CT perfusion or collateral assessment were used in all patients. This is a 54% increase in our previous estimate in 2021. Over 50% of LAO strokes are now potentially eligible for thrombectomy. The increase in eligibility is principally due to a much larger cohort of later presenting and/or larger ischaemic core patients.

**Conclusion:**

Most previously independent LAO stroke patients presenting within 24 h, even in the presence of a large ischaemic core on initial non-contrast CT, should be considered for thrombectomy with use of advanced brain imaging in those presenting beyond 12 h to identify salvageable penumbral brain tissue. Treatment in most patients remains critically time-dependent and our estimates should be interpreted with this in mind.

## Graphical abstract

**Graphical abstract:**
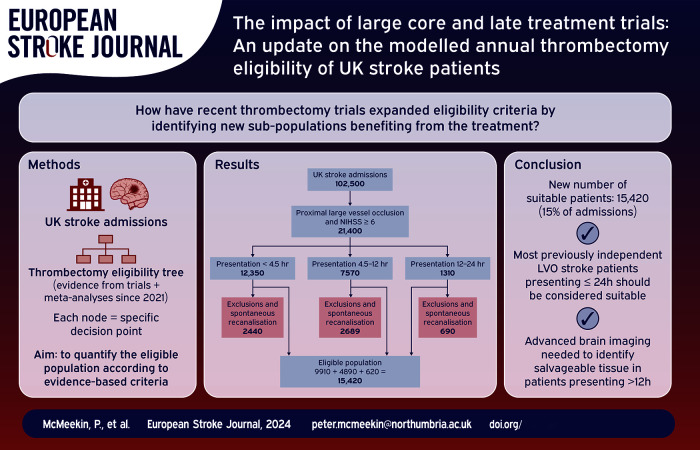

## Background and aims

Thrombectomy for selected patients with large artery occlusive (LAO) stroke is a highly effective treatment^1^ with a rapidly progressing evidence-base. Expansion of the eligible population creates challenges for the planning and resourcing of clinical services. Decision tree modelling can provide the best available information to assist with understanding the volume of diagnostics and treatments required now and in the near future.

In 2017 we modelled demand based upon evidence from eight randomised controlled trials (RCTs) comparing thrombectomy to best medical care: ESCAPE^2^; SWIFT PRIME^3^; REVASCAT^4^; MR CLEAN^5^; EXTEND-IA^6^; THRACE^7^; PISTE^8^ and THERAPY.^9^ We incorporated evidence from these trials with parameters from registries and other epidemiological studies to estimate that in the United Kingdom approximately 10,000 stroke admissions per year (10% of all stroke admissions) would be eligible for thrombectomy.^10^

By 2021, further trials provided evidence that extended the eligibility for thrombectomy. DAWN^11^ and DEFUSE 3^12^ showed that thrombectomy was effective beyond the time window specified in the original trials that informed our 2017 estimate. Along with ESCAPE, RESILIENT,^13^ POSITIVE^14^ and REVASCAT, they were included in the AURORA meta-analysis,^15^ which evidenced the effectiveness of thrombectomy beyond 6 h for patients with salvageable brain tissue. Manceau et al. reported that patients with larger core infarcts (DWI-ASPECTS < 5) could also benefit.^16^ However, after including the totality of the updated evidence, the estimated number of UK patients eligible for thrombectomy did not change appreciably from that first estimated in 2017.^17^ However, we noted then that because of the global COVID pandemic the results of several ongoing important trials were delayed.

By October 2023, a further eight of these trials had published results relevant to new sub-populations of LAO stroke patients including those who have vertebrobasilar occlusions, present later, or have larger core infarcts/worse ASPECTS (BEST,^18^ BASICS,^19^ ATTENTION,^20^ BAOCHE,^21^ RESCUE Japan LIMIT,^22^ ANGEL-ASPECTS,^23^ MR CLEAN LATE,^24^ TENSION^25^ and SELECT2^26^). Therefore, we have updated our 2021 estimates incorporating evidence about eligibility based on benefit in these new sub-populations of stroke patients. As with our 2021 estimates, we have also considered the implications for UK services and our results also serve as an indicator for other comparable countries.

## Methods

To produce our updated estimates of UK annual mechanical thrombectomy eligibility, we further developed our eligibility tree approach as previously described but remained consistent with the methodology used to derive previous estimates.^10,17^ This involved reviewing thrombectomy trials and meta-analyses reporting since our last update and searching the published evidence base for relevant information. In the tree, each node describes a decision point and quantifies the eligible/ineligible population according to specific criteria. The root node of the eligibility tree contains the annual UK stroke hospital admissions incidence and terminates with the subset of patients eligible for thrombectomy.

The decision criteria are derived from the highest level of evidence available (wherever possible individual patient data meta-analysis of multiple RCTs or, failing that, extraction of information from multiple individual RCTs). For a node where no specific RCT data existed, evidence was obtained from the latest systematic review(s) or failing that, the highest quality relevant observational data available – such as prospective national audit data. To revise our tree, we reviewed the evidence up to the end of October 2023 for each node and, if necessary, revised its structure to reflect new or updated knowledge and patient selection criteria. Two authors (PM & PW) independently searched 2021 to October 2023 for the evidence relevant to decision nodes B to N. No exclusion from eligibility is made on the grounds of geography or lack of 24 h thrombectomy service provision.

The review process involved the authors discussing the quality of the evidence and its’ applicability to the United Kingdom and generalisability. Three experienced clinical authors (GAF, MJ, PMW) assessed the evidence to reach a consensus decision on inclusion. Where differences of opinion arose around the generalisability or applicability of data, these were resolved by discussion and agreeing a consensus. The evidence identified supporting updates for each node and the decision consequences are described in the Results.

## Results

**Figure 1.**
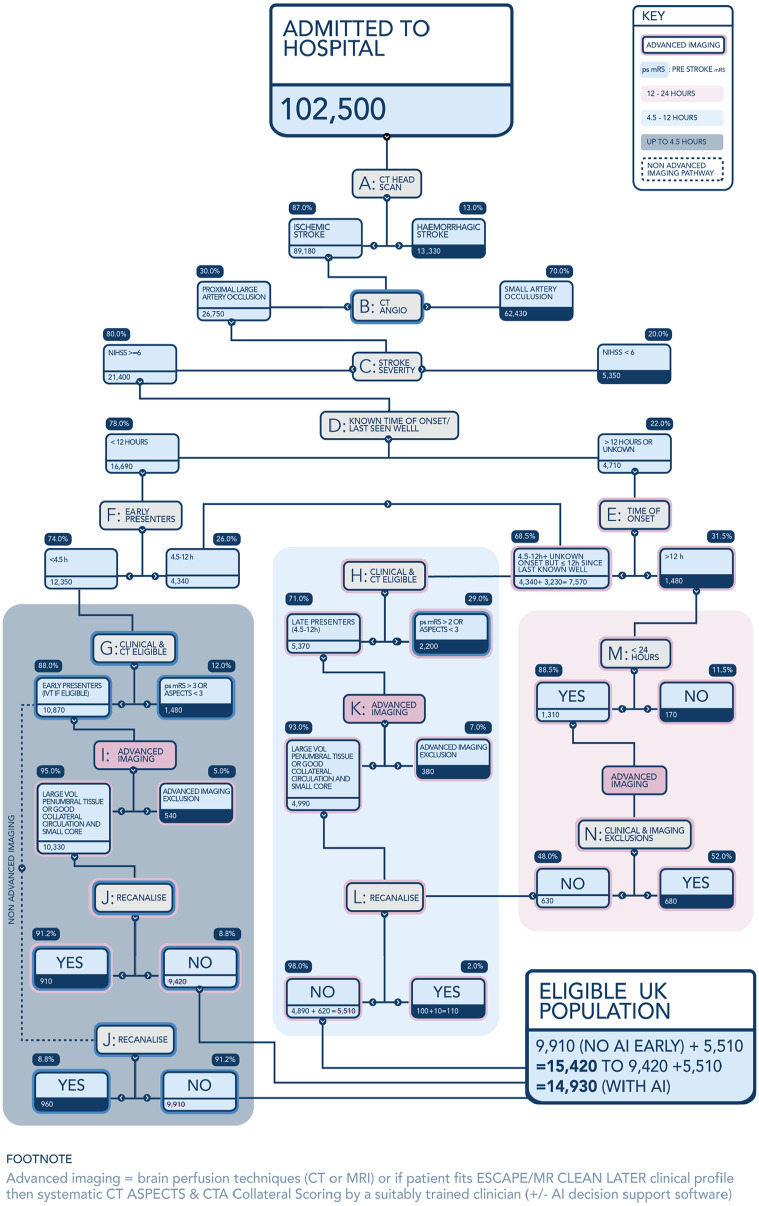
Eligible UK population including.

The eligibility tree begins with the annual number of stroke hospital admissions in the United Kingdom (population 67.7 million), which is refined first by excluding haemorrhagic and then non-LAO ischaemic strokes that are not caused by large artery occlusion (LAO) – nodes A and B (Figure 1). The UK incidence of acute stroke admissions has increased appreciably since 2017 from 95,500 to 102,419. Our assessment that 87% of all stroke admissions are ischaemic is unchanged. This is based on the Stroke Sentinel National Audit Programme (SSNAP), which includes all patients admitted to hospitals within England, Wales and Northern Ireland^27^ and the Scottish Stroke Care Audit for patients admitted to hospitals in Scotland (population 5.2 million).

The first updated eligibility parameter is at node B, where CT (or MR) angiography identifies the presence and location of LAO. Originally, in 2017, an incidence of 40% was based on the EXTEND IA trial^6^ and the Screening Technology and Outcome Project in Stroke Study (STOP-Stroke)^28^ supported by a then-recent UK study.^29^ Our 2021 update revised this down to 35% based on a meta-analysis by Rennert et al.^30^ The most recent 2023 evidence review identified two further studies that supported further reducing this proportion. Lakomkin et al. systematically reviewed these along with 14 other studies in a meta-analysis, which weighted their reported incidence by study size, giving an incidence of LAO ranging from 29.3% to 31.1%.^31^ Much of the reduction in estimated LAO incidence is due to the revised and more precise definition of LAO now used. Specifically, we now exclude distal arterial occlusions not readily accessible with current thrombectomy devices or medium sized arterial occlusions. The prior 35% estimate of Rennert et al. included these occlusions, where the benefit of thrombectomy is, as yet, unproven. We therefore revise our estimate of the incidence of proximal arterial occlusion (including proximal M2 MCA) in which Thrombectomy is of proven efficacy to 30%. This systematic review-based decision was additionally supported by evidence from a recent population-based study in France reporting an (unadjusted) incidence of LAO of 29%.^32^ This revision reduces the numbers of patients entering node C by 8% from 29,100 to 26,750 despite the increase in overall stroke admissions.

The proportion of LAO stroke patients with a National Institute for Health Stroke Scale (NIHSS) score of 6 or more remains at 80% (node C). We identified no new evidence to indicate that the previous estimates of stroke severity should be updated, although we note that thrombectomy trials in mild stroke with a low NIHSS score are still ongoing. Although the MR CLEAN LATE trial^24^ included a modest number of patients with NIHSS < 6 (we estimate around 105 subjects), the ‘low NIHSS’ (0–6) subgroup as published^24^ still included NIHSS of 6 and the confidence intervals for this subgroup crossed the non-significant odds ratio threshold (of 1.0); nor did the investigators provide data on screening logs to afford an estimate of the proportion of LAO strokes with low NIHSS to impact our node C denominator. All other recent trials have only included those patients with NIHSS ⩾ 6.

Also unchanged are the estimates for the proportion of patients presenting with a known time of onset within 12-h (78%; node D), the proportion of those arriving after 12 h with a known time of onset (31.5%; node E) and the proportion of those presenting within 12 h but within the updated potential 9-h time window for intravenous thrombolysis (IVT) (74%; node F).

At this level the thrombectomy eligibility decision tree has three branches beginning at nodes G, H and M:

- 12,350 early presenters <4.5 h (within the conventional IVT window) entering node G.
- 7570 patients entering node H who presented with known time of onset 4.5–12 h or who were last known well within 12 h.
- 1480 patients entering node M with a known time of onset more than 12 h or who were last known well >12 h prior to arrival at hospital.

The remainder of the eligibility decisions consider the clinical and radiological criteria specific to each subgroup, updated by new randomised trial evidence published since 2021.

Nodes G, I and J together consider early presenters who are potentially suitable for thrombolysis. Node G recognises the combined clinical and imaging criteria for MT. The criteria for early thrombectomy are: a pre-stroke (estimated) modified Rankin Scale score of 0–3 combined with CT (or MRI) imaging showing an Alberta stroke programme early CT score (ASPECTS) of >2. The most recent SSNAP data (2022-23) SSNAP Annual Report indicates that just 8% of patients arriving within this 4.5 h time window had a pre stroke (ps) mRS of 4 or 5 .^27^ It is also clear from national audit data that in the UK LAO stroke patients with a pre stroke mRS of 3 are being offered MT routinely in some centres; so we therefore did not exclude them from eligibility. Indeed, a small number of mRS ⩾ 3 participants were included in the MR CLEAN (4%) & MR CLEAN LATE (2%) trials.^5,24^ Since our previous 2021 estimate, the SELECT 2, ANGEL ASPECTS, RESCUE Japan LIMIT & TENSION trials have shown benefit from MT in patients with an ASPECTS of 3–5.^23,25,26,32^ Consequently, the criteria at node G are adjusted to only exclude patients with an ASPECTS of 2 or less, whereas previously ASPECTS 3 and 4 were excluded. This increases MT eligibility at node G from 71% to 88%. Based on SSNAP data and through consensus review of literature, it was agreed that it can be predicted that ~5% of patients would have an ASPECTS value of 2 or worse and that this value should be added to the pre stroke mRS 4–5 exclusions, but that the total should be marginally reduced (from 13% to 12%) to allow for some potential overlap between the two criteria. The updated tree estimates that 10,870 patients leave node G (i.e. 1350 additional patients) and proceed down the eligibility tree to either advanced imaging or direct to thrombectomy.

The <4.5 h subgroup proportions excluded by advanced brain imaging and who spontaneously recanalise remain unchanged at 5% (node I) and 9%^33^ (node J) respectively. The former estimate is unchanged and is based on data from EXTEND-IA trial and the Sistema Online d’Informació de l’Ictus Agut (SONIIA) Registry indicating that advanced brain imaging (CT Perfusion or MRI techniques) only excludes a minority of early presenters (>4.5 h).^6,34^ After these exclusions, the updated tree indicates 9420 early presenters eligible for thrombectomy if advanced brain imaging is used to exclude patients without a reasonable volume of penumbral tissue, or 9910 eligible if advanced imaging is not used. These represent a 14% increase from our previous estimates of 8300 and 8700 respectively.

The 9050 patients in the remaining two branches of the eligibility tree are those most affected by the new evidence available since 2021, namely: (i) 7570 patients presenting with an unknown time of onset or who were last known well (LKW) within 12 h or with a known time onset 4.5–12 h and (ii) 1480 patients with presentations >12 h since onset/LKW. This new evidence comes from seven trials, four of which relate to posterior circulation strokes: BEST,^18^ BASICS^19^i, ATTENTION^20^ and BAOCHE.^21^ Whilst the results of the earlier BEST and BASICS trials were inconclusive, ATTENTION and BOACHE demonstrated the benefit of thrombectomy over best medical treatment (BMT) alone for patients with an intracranial vertebral or a basilar-artery occlusion and who have a moderate-to-severe neurological deficit (NIHSS of 10 or more) and limited early ischaemic changes up to 24 h after stroke onset. The benefit of thrombectomy for LAO patients with larger regions of anterior circulation infarction (core) up to 24 h after stroke onset was demonstrated by RESCUE-Japan LIMIT,^22^ SELECT2^26^ ANGEL-ASPECTS^23^ and TENSION.^25^ MR CLEAN LATE demonstrated the benefits of thrombectomy 6–24 h over BMT after onset for patients with early infarct changes in less than a third of the corresponding middle cerebral artery territory and adequate collateral flow identified by CTA.^24^

Previously we had excluded 72.5% of patients at node H (4.5–12 h of known time onset or LKW 4.5–12 h prior to admission), because of clinical (mRS > 2) and imaging (ASPECTS of 5 or less) criteria. Robust evidence of thrombectomy benefit for ASPECTS of 3–5 in this time window means that the estimate for patients rendered ineligible by low ASPECTS alone drops markedly to 11%. This figure was derived from the trials of thrombectomy in the 6–24-h time period since stroke onset, which combined report an average of 11% of patients with ASPECTS 0–2. From SSNAP data an unchanged 19% of LAO patients have a pre-stroke mRS of 3 or more. This modification to imaging eligibility more than doubles the estimate of the numbers eligible at node H from 2300 to 5370. Of those moving on to advanced brain imaging (node K), 43% patients were previously excluded from eligibility based upon a core volume of >70 mL whereas we now exclude only 7% of patients at node K. This reflects revised exclusion criteria regarding perfusion imaging to only exclude those on perfusion with penumbral volume <15 mL or alternatively those with CTA collateral score of zero (on the 4 point Tan scale of 0–3^35^). The DEFUSE 3 trial reported that 7% of participants had a mismatch volume of <15 mL and this threshold has been incorporated into the 2023 National Clinical Guideline for Stroke for the UK and Ireland.^36^ An observational study of automated CT classification software interrogating CT scans from 98 consecutive patients potentially eligible for thrombectomy described that 93% of (LAO) patients had a collateral score on the Tan scale of 1 or more.^37^ In practice, because usually either the CTA_CS_ + ASPECTS or CTP (or MRI) imaging criterion, but not both, are used in triage, we chose not to inflate the imaging exclusion estimate beyond 7%. The result of this is that only 380 patients are excluded at node K compared to 1000 in our previous model.

For pre thrombectomy recanalisation in later presenters (node L), we retained an unchanged rate of 2% amongst both the 4.5–12 h & 12–24 h populations. We recognise that there will be a small but probably increasing number of patients who receive thrombolysis based upon advanced imaging within the 4.5–9 h (known onset) or last seen well 4.5–24 h time windows, which could increase the rate of recanalisation within this group. However, the recanalisation effect of IVT in the late time window may well be lower, and we have left the 2% rate unchanged until more data on both late IVT rates and efficacy become available. Thus, the changes to clinical and imaging exclusion criteria and advanced imaging selection criteria mean that despite reduced estimates of LAO incidence, there are 4890 UK LAO stroke patients presenting within 4.5–12 h of onset/LKW who are now eligible for thrombectomy (with most not being eligible for thrombolysis). We previously estimated this figure to be 1250, that is, an additional 3640 eligible.

The final branch of the MT eligibility tree contains patients who present between 12 and 24 h after stroke onset. The number of patients falls from 1500 to 1400 at node M after those presenting after 24 h are excluded, based on SSNAP data that indicates that 11.5% of ischaemic stroke patients with LAO present >24 h.^27^ The revised clinical/imaging exclusion rate in this group at node N is 52%. This is derived as follows: 30% of patients in this time window have a pre-stroke mRS of 2 or more,^27^ 15% of late time window patients have an ASPECTS value of 2 or lower and 7% have a perfusion mismatch of >15 mL (from ANGEL-ASPECTS trial logs^23^). Nevertheless, compared to a previous eligibility at node N of 5% based upon more restrictive DAWN trial criteria,^11^ there is a considerable increase in the patients eligible for MT within the 12–24 h time window from 70 to 630 before taking account of pre-procedural recanalisation.

The TESLA trial included patients with CT ASPECTS 2–5 but is modest sized and although it has reported in brief (ESOC Munich 2023), it has not yet been published to ascertain whether there are sufficient ASPECTS 2 patients (based on core lab review) enrolled 6–24 h to routinely extend eligibility from ASPECTS 3–5 to 2–5. Provisional TESLA results were however included in a recent meta-analysis which confirmed the benefits of thrombectomy in larger ischaemic core patients.^38^ The LASTE^39^ trial along with TESLA might provide enough data to change ASPECTS 2 eligibility in practice, but as a single small medium trial with most recruits in ASPECTS 2–5 category, LASTE very likely won’t change practice regarding ASPECTS 0–1 on its own.

## Discussion

Re-evaluation of the thrombectomy evidence and stroke incidence in mid-2023 shows significant changes to the UK population of stroke patients eligible for thrombectomy relative to the 2021 update of our original 2017 estimate. This is principally the result of the inclusion of a much larger cohort of late-presenting and larger ischaemic core patients, compared with the previous evidence. Interestingly, the UK incidence of acute stroke admissions has increased appreciably since 2017 from 95,500 to 102,419 in 2022. In our 2021 update, we had incorporated an estimated incidence of 120,000 by 2030 and these updated figures confirm this predicted trend.

Overall, we now estimate that 15,420 (approximately 15%) of stroke patients admitted to hospitals in the UK would be eligible for thrombectomy or 14,930 if advanced brain imaging were used in all patients. Previously we estimated a lower figure of 10,020 (9580 if Advanced Imaging were used) – so an absolute increase of 5400 patients (a relative increase of 54%). It also means that of just under 27,000 UK admissions per annum with LAO stroke, 58% should now be considered eligible for thrombectomy. This change to ‘*more likely eligible than not*’ has important implications for imaging and hyperacute stroke resourcing, particularly considering the historically low level of provision of ischaemic stroke neurointervention in the UK (and elsewhere).

Of the three temporal subgroups, those presenting between 4.5 and 12 h and those presenting between 12 and 24 h saw the biggest absolute and proportionate increases respectively that is, 1250–4890 for 4.5–12 h and 70–617 for the 12–24 h group. Although the great majority of additional eligible patients (4257/5400) are in the late-presenting 4.5–24 h groups, treatment in most remains critically time-dependent, so this cannot be viewed as an excuse to take up to 24 h to deliver MT if the population benefits promised by the RCTs are to be realised. For instance, the MR CLEAN LATE trial showed a clear trend in a reducing Odds Ratio for benefit from thrombectomy in the later onset to randomisation subgroups, with only the earliest 5.8–9.8 h subgroup demonstrating a statistically significant benefit from thrombectomy on its own, even though the 3 temporal subgroups were similarly sized.

Any increase in eligibility for thrombectomy should translate into better outcomes, but there are some caveats. Excepting the highly selected DAWN/DEFUSE-3 populations, absolute mRS independent outcomes (0–2) for thrombectomy in the large core stroke presenters in the new trials are rather modest at an aggregate of 21% [Japan RESCUE, ANGEL ASPECTS, SELECT 2, TENSION] compared with the independent outcome rate in the 7 HERMES trials – an mRS 0–2 of 48% at 90 days.^1,7,8^ Even with small core infarcts, the outcome in the later time window less selective, late-presenting population is worse – MR CLEAN LATE (with median ASPECTS of 9) demonstrated a mRS 0–2 rate of 39% in the thrombectomy arm. The recent late window/larger core trials are also associated with higher mortality than early time window (HERMES) trials – an aggregate mortality of 29% in the EVT arms of 4 recent large core trials and 24% in MR CLEAN LATE versus 15% in HERMES. Thus, time to treatment remains critical in maximising both the individual benefit and the cost-effectiveness of thrombectomy.

Given the greatly increased eligibility rates between 4.5–12 h and 12–24 h there is a need for advanced brain imaging to be routinely available to many more patients than has been the case until now, in the UK at least, including for the significant proportion of patients presenting with wake-up stroke (12%) or with no known onset time (31%).^27^ Ideally, we should be replicating the imaging-based selection methods used in the trials that showed the benefit. Independent evaluation of novel AI software tools linked to reperfusion decision making should be an area of urgent research examining their impact on ultimate clinical outcomes, particularly as both MR CLEAN LATE^24^ and TENSION^25^ were able to demonstrate significantly better outcomes with thrombectomy without the use of CT perfusion and AI-based processing software.

The recent RCTs informing this updated MT eligibility tree overwhelmingly excluded acute stroke patients with any pre-stroke disability (pre stroke mRS of 2 or more). Around a third of UK stroke admissions have pre-existing disability (mRS 2 or more) and our methodology may underestimate the extent to which MT is offered to these patients. Frailty, which is distinct from but often coexists with pre-stroke disability, may be another factor in clinicians’ decision making, yet no trials have reported on pre-stroke frailty, and we were unable to include this in our eligibility tree. Finally, cost-effectiveness of thrombectomy with large core or with pre-stroke disability especially in later time windows is likely to be less clear cut due to lower proportions of patients achieving a very good/independent recovery. Indeed publicly funded healthcare systems such as the UK National Health Service might consider limiting access for late-window, larger core patients if the cost-effectiveness is evaluated as ‘unfavourable’ (e.g. >£20,000 per Quality Adjusted Life Year gained).

The estimated volume of treatments for the UK can only be achieved if there is adequate clinical infrastructure and efficient emergency care pathways to identify and rapidly transport appropriate patients towards thrombectomy providers. The collection of national audit data and involvement of regional networks supports the continuous review of service performance whilst ongoing studies are evaluating both enhanced communication between paramedics and comprehensive stroke centres^40^ and point of care diagnostics^41^ which could select patients for direct admission to thrombectomy centres and reduce delays incurred during secondary transfer. Such developments would be particularly important because stroke incidence is likely to rise further than already seen in the UK between 2017 and 2022 (>7% absolute increase), primarily due to an ageing population. Additionally, low NIHSS and/or medium vessel occlusion patients may in the near future be shown to benefit from thrombectomy. Therefore, it is predictable that the need for thrombectomy will rise substantially, with perhaps >20% of stroke patients ultimately eligible.

We estimate eligibility for all populations of the UK regardless of geography because more than 95% of people live within 90 min travel by ground-based ambulance of a Neuroscience Centre and 99% live within 120 min [REF- Allan M et al. modelling MT access]. There is also evidence that for the most remote populations helicopter secondary transfer is clinically and cost effective.^42^

It is important to recognise limitations in the decision tree that could impact upon its’ accuracy and generalisability. Wherever possible, we used parameters from RCTs and larger observational/non-randomised studies, which were already referenced within national clinical guidelines. It was occasionally necessary for the authors to adopt a consensus approach for factors with lower levels of evidence, such as the interaction between dependency and ASPECTS scores. It is important that trials and registries continue to share descriptive data and screening logs for inclusion in future modelling.

## Conclusion

Modelling the implications of recently published RCTs in combination shows a substantial 54% increase in the proportion of UK stroke patients eligible for thrombectomy. As a result, 15% of all stroke admissions and nearly 60% of LAO patients should now be eligible. Most previously independent LAO stroke patients presenting within 24 h even in the presence of a large ischaemic core on plain CT, should be considered for thrombectomy and most of those presenting beyond 12 h will require advanced brain imaging to identify salvageable penumbra. These substantial increases in eligibility will present challenges in the UK with its relatively underdeveloped imaging and neurointerventional infrastructure and low proportion of acute stroke centres with thrombectomy capability. However, our findings have wider applicability than the UK; with a current European average thrombectomy rate of 6.3%^43^ this represents a universal challenge for most healthcare systems if we are to deliver the population benefits from reduced disability in major stroke that are offered by the latest trials in later-presenting and/or large infarct core patients.
